# Drug retention and discontinuation reasons between seven biologics in patients with rheumatoid arthritis -The ANSWER cohort study-

**DOI:** 10.1371/journal.pone.0194130

**Published:** 2018-03-15

**Authors:** Kosuke Ebina, Motomu Hashimoto, Wataru Yamamoto, Akira Ohnishi, Daijiro Kabata, Toru Hirano, Ryota Hara, Masaki Katayama, Shuzo Yoshida, Koji Nagai, Yonsu Son, Hideki Amuro, Kengo Akashi, Takanori Fujimura, Makoto Hirao, Keiichi Yamamoto, Ayumi Shintani, Atsushi Kumanogoh, Hideki Yoshikawa

**Affiliations:** 1 Department of Orthopaedic Surgery, Osaka University, Graduate School of Medicine, Osaka, Japan; 2 Department of Advanced Medicine for Rheumatic Diseases, Graduate School of Medicine, Kyoto University, Kyoto, Japan; 3 Department of Health Information Management, Kurashiki Sweet Hospital, Kurashiki, Japan; 4 Department of Rheumatology and Clinical Immunology, Kobe University Graduate School of Medicine, Kobe, Japan; 5 Department of Medical Statistics, Osaka City University Graduate School of Medicine, Osaka, Japan; 6 Department of Respiratory Medicine and Clinical Immunology, Osaka University, Graduate School of Medicine, Osaka, Japan; 7 The Center for Rheumatic Diseases, Department of Orthopaedic Surgery, Nara Medical University, Nara, Japan; 8 Department of Rheumatology, Osaka Red Cross Hospital, Osaka, Japan; 9 Department of Internal Medicine (IV), Osaka Medical College, Osaka, Japan; 10 First Department of Internal Medicine, Kansai Medical University, Osaka, Japan; 11 The Center for Rheumatic Diseases, Nara Medical University, Nara, Japan; Charles P. Darby Children's Research Institute, UNITED STATES

## Abstract

The purpose of this study was to evaluate the retention and discontinuation reasons of seven biological disease-modifying antirheumatic drugs (bDMARDs) in a real-world setting of patients with rheumatoid arthritis (RA). 1,037 treatment courses with bDMARDs from 2009 to 2016 [female, 81.8%; baseline age, 59.6 y; disease duration 7.8 y; rheumatoid factor positivity 81.5%; Disease Activity Score in 28 joints using erythrocyte sedimentation rate (DAS28-ESR), 4.4; concomitant prednisolone 43.5% and methotrexate 68.6%; Bio-naïve, 57.1%; abatacept (ABT), 21.3%; tocilizumab (TCZ), 20.7%; golimumab (GLM), 16.9%; etanercept (ETN), 13.6%; adalimumab (ADA), 11.1%; infliximab (IFX), 8.5%; certolizumab pegol (CZP), 7.9%] were included in this multi-center, retrospective study. Drug retention and discontinuation reasons at 36 months were estimated using the Kaplan-Meier method and adjusted by potent confounders using Cox proportional hazards modeling. As a result, 455 treatment courses (43.9%) were stopped, with 217 (20.9%) stopping due to inefficacy, 113 (10.9%) due to non-toxic reasons, 86 (8.3%) due to toxic adverse events, and 39 (3.8%) due to remission. Drug retention rates in the adjusted model were as follows: total retention (ABT, 60.7%; ADA, 32.7%; CZP, 43.3%; ETN, 51.9%; GLM, 45.4%; IFX, 31.1%; and TCZ, 59.2%; P < 0.001); inefficacy (ABT, 81.4%; ADA, 65.7%; CZP, 60.7%; ETN, 71.3%; GLM, 68.5%; IFX, 65.0%; and TCZ, 81.4%; P = 0.015), toxic adverse events (ABT, 89.8%; ADA, 80.5%; CZP, 83.9%; ETN, 89.2%; GLM, 85.5%; IFX, 75.6%; and TCZ, 77.2%; P = 0.50), and remission (ABT, 95.5%; ADA, 88.1%; CZP, 91.1%; ETN, 97.5%; GLM, 94.7%; IFX, 86.4%; and TCZ, 98.4%; P < 0.001). In the treatment of RA, ABT and TCZ showed higher overall retention, and TCZ showed lower inefficacy compared to IFX, while IFX showed higher discontinuation due to remission compared to ABT, ETN, GLM, and TCZ in adjusted modeling.

## Introduction

Biological disease-modifying antirheumatic drugs (bDMARDs) have dramatically improved the management of rheumatoid arthritis (RA). Tumor necrosis factor inhibitors (TNFi) were the first bDMARDs used for RA, and abundant evidence has been accumulated regarding the efficacy, safety, and tolerability of adalimumab (ADA), etanercept (ETN), and infliximab (IFX) [1–5]. On the other hand, other TNFi such as golimumab (GLM) (2011) and certolizumab pegol (CZP) (2013) were lately licensed for RA in Japan. In addition, the European League against Rheumatism (EULAR) announced a 2013 recommendation regarding the management of RA with bDMARDs, in which tocilizumab (TCZ) and abatacept (ABT) were also considered as efficacious and safe as TNFi, which should be considered as a first-line biologic agent [6]. However, clinicians’ choice of bDMARDs may depend on various factors (patients’ background characteristics such as age, comorbidities, combined conventional synthetic DMARDs (csDMARDs), previously administered bDMARDs, economic burden, etc.) in clinical practice, and reliable selection criteria for these bDMARDs are still lacking.

The adaptive criterion of randomized controlled trials (RCTs) is sometimes limited to patients who are quite different from those in real-world settings [7], and observational studies of cohort-based registries have increasingly been used to investigate the performance of bDMARDs [1–4, 8–10]. In addition, drug retention in observational studies can be considered as a composite measure and index of effectiveness, safety and tolerability [4, 11–13]. On the other hand, treatment selection and discontinuation may be influenced by factors such as differences in patient characteristics and attending physicians in observational studies, although multi-center studies and the national health insurance in our country may help to diminish these possible deviations [11–13].

The aim of this multi-center, retrospective study was to clarify the retention and reasons for discontinuation of seven biologics in the real-world setting of RA.

## Materials and methods

### Patients

The Kansai Consortium for Well-being of Rheumatic Disease Patients (ANSWER) cohort is an observational multi-center registry of patients with RA in the Kansai district of Japan. Data of patients at seven institutes (Kyoto University, Osaka University, Osaka Medical College, Kansai Medical University, Kobe University, Nara Medial University, and Osaka Red Cross Hospital) were included. From 2011 to 2016, 4,461 patients with RA ≥20 years were registered, and 52,654 serial disease activities were available from the database. Data from patients with RA treated using one of seven bDMARDs (ABT, ADA, CZP, ETN, GLM, IFX, and TCZ; including both intravenous and subcutaneous agents, but excluding bio-similar agents, all of which were introduced between January 2009 and September 2016) were retrospectively collected. All patients with RA fulfilled the 1987 classification criteria of the American College of Rheumatology [14], and also had full baseline demographic data such as age, sex, disease activity (Disease Activity Score in 28 joints using erythrocyte sedimentation rate [DAS28-ESR]), disease duration of RA, number of previously administered bDMARDs, reasons for discontinuation of bDMARDs, dates of both starting and discontinuing bDMARDs, concomitant doses of MTX and PSL, and presence of other csDMARDs for which evidence has been accumulated to enhance the efficacy of bDMARDs, such as bucillamine (BUC) [15, 16], iguratimod (IGU) [17], salazosulfapyridine (SASP) [16, 18], and tacrolimus (TAC) [19, 20]. Patients without data for these parameters were excluded. Other baseline demographic features such as rheumatoid factor (RF) and anti-cyclic citrullinated peptide antibody (ACPA) positivity, and Health Assessment Questionnaire [HAQ] disability index [DI] score were also collected.

Treatments were administered by the attending rheumatologists in accordance with guidelines of the Japan College of Rheumatology. Drug retention was retrospectively evaluated as the duration until definitive treatment interruption. Reasons for discontinuation of biologics were analyzed and classified into four major categories: 1) inefficacy (including primary and secondary); 2) remission; 3) toxic adverse events (infection, skin or systemic reaction, and other toxic events [including hematologic, pulmonary, renal, cardiovascular complications and malignancies]; and 4) nontoxic reasons (patient preference, change in hospital, desire for pregnancy, etc.). Physicians were allowed to cite only one reason for discontinuation. The representative facility of this registry is Kyoto University, and this observational study (not clinical trial) was conducted in accordance with the Declaration of Helsinki (results are not published elsewhere), and approved by each ethics committee of seven institutes (Kyoto University, Osaka University, Osaka Medical College, Kansai Medical University, Kobe University, Nara Medial University, and Osaka Red Cross Hospital). In addition, the detail of this study is shown in the homepage of Osaka University Graduate School of Medicine (approval number; 15300), and written informed consent was obtained from all participants prior to enrollment.

### Statistical analysis

Baseline characteristics were compared across the seven bDMARDs. The significance of differences was assessed using the Kruskal-Wallis nonparametric test for continuous variables and Pearson’s chi-square test for categorical variables. The survival curves of each biologic explained by specific causes were examined by the Kaplan-Meier method and compared statistically using a stratified log-rank test. The time to discontinuation of biologics was analyzed using Cox proportional hazards modeling [1]. The proportion of treatment discontinuation explained by specific causes were analyzed at 36 months, and also adjusted by potential confounders that may influence drug discontinuation and the incidence of adverse events, as previously described (sex, baseline age, disease duration, DAS28-ESR, HAQ-DI, RF and ACPA positivity, concomitant MTX and PSL dose, presence of concomitant csDMARDs (BUC, IGU, SASP, and TAC), date of starting bDMARDs, and number of previously administered bDMARDs) [1, 8–10, 21]. Multivariate Cox proportional modeling was designed using stepwise backward deletion in choosing those covariates showing values of P < 0.05 for adjustment.

Statistical analyses were performed using EZR (Saitama Medical Center, Jichi Medical University, Saitama, Japan), a graphical user interface for R (The R Foundation for Statistical Computing, Vienna, Austria) [22]. P < 0.05 were considered statistically significant.

## Results

### Baseline characteristics

The study population was selected from all patients with RA in the ANSWER cohort (n = 4461) who fulfilled the inclusion criteria (n = 750; 1037 bDMARD treatment courses). Baseline demographic and clinical characteristics of the enrolled patients (ABT, n = 221; ADA, n = 115; CZP, n = 82; ETN, n = 141; GLM, n = 175; IFX, n = 88; TCZ, n = 215) are described in Table 1. Overall at baseline, mean age was 59.6 years, 81.8% of participants were female, mean disease duration was 7.8 years, RF positivity was 81.5%, ACPA positivity was 86.7%, mean DAS28-ESR score was 4.4, and mean HAQ-DI score was 1.1. In addition, concomitant medications were PSL in 43.5%, MTX in 68.6%, SASP in 23.3%, BUC in 10.0%, TAC in 6.8%, and IGU in 2.0%. The bDMARD being administered was the first in 57.1%, second in 24.0% and third or more in 18.9%.

**Table 1:** Clinical characteristics at initiation of each biologic agent.

Variable	ABT (n = 221)	ADA (n = 115)	CZP (n = 82)	ETN (n = 141)	GLM (n = 175)	IFX (n = 88)	TCZ (n = 215)	P-value
Age (years)	64.4±11.7	55.1±12.8	56.4±17.1	58.6±15.2	61.4±14.3	55.3±13.2	58.9±14.1	<0.001
Female sex (%)	80.5	78.3	87.8	85.1	87.4	76.1	78.1	0.067
Disease duration (years)	8.9±10.1	4.0±5.9	5.8±8.0	8.4±10.2	10.7±11.4	3.6±6.4	8.8±9.0	<0.001
RF positivity (%)	87.2	79.3	83.3	79.8	83.8	73.2	79.1	0.2
ACPA positivity (%)	88.1	88.2	88.4	89.1	85.3	81.7	85.9	0.77
DAS28-ESR	4.4±1.3	4.2±1.2	4.6±1.5	4.4±1.5	4.3±1.3	4.5±1.6	4.6±1.5	0.19
HAQ-DI	1.2±0.8	0.9±0.7	1.1±0.9	0.9±0.8	1.1±0.9	1.1±0.9	1.2±0.8	0.16
PSL usage (%)	48.4	33	45.1	41.1	42.3	34.1	49.8	0.025
PSL dose (mg/day)	3.2±6.9	1.7±3.1	2.8±4.2	2.2±3.3	2.1±2.9	1.6±2.7	3.1±4.1	0.005
MTX usage (%)	56.1	89.6	72	59.6	70.9	95.5	61.9	<0.001
MTX dose (mg/week)	4.4±4.5	8.5±4.1	6.4±4.8	5.2±4.8	5.9±4.5	8.9±4.0	5.2±4.7	<0.001
SASP usage (%)	33.5	22.6	25.6	22.7	24.6	13.6	15.8	<0.001
BUC usage (%)	14.9	7.8	4.9	10.6	12.6	4.5	7.9	0.027
TAC usage (%)	14.5	2.6	4.9	2.1	5.1	3.4	7.4	<0.001
IGU usage (%)	1.8	0.9	1.2	2.1	2.3	1.1	3.3	0.89
1^st^ bio (%)	63.8	77.4	58.5	63.1	41.1	83	37.2	<0.001
2^nd^ bio (%)	19.9	16.5	15.9	20.6	35.4	10.2	34	<0.001
≧3rd bio (%)	16.3	6.1	25.6	16.3	23.5	6.8	28.8	<0.001

Values represent mean ± standard error (SE), unless otherwise noted. ABT = abatacept, ADA = adalimumab, CZP = certolizumab pegol, ETN = etanercept, GLM = golimumab, IFX = infliximab, TCZ = tocilizumab, RF = rheumatoid factor, ACPA = anti-cyclic citrullinated peptide antibody, DAS28-ESR = Disease Activity Score in 28 joints using erythrocyte sedimentation rate, HAQ-DI = Health Assessment Questionnaire disability index, PSL = prednisolone, MTX = methotrexate, SASP = salazosulfapyridine, BUC = bucillamine, TAC = tacrolimus, IGU = iguratimod. Bio = biologic agent.

The significance of differences was assessed using the Kruskal-Wallis nonparametric test for continuous variables and Pearson’s chi-square test for categorical variables.

Between the seven bDMARDs, no significant differences were observed in baseline sex, RF or ACPA positivity, DAS28-ESR, or HAQ-DI. On the other hand, significant differences were observed in baseline age (P < 0.001), disease duration (P < 0.001), PSL usage (%) (P = 0.025), PSL dose (mg/day) (P = 0.005), MTX usage (%) (P < 0.001), MTX dose (mg/week) (P < 0.001), SASP usage (%) (P < 0.001), BUC usage (%) (P = 0.027), TAC usage (%) (P < 0.001), and number of previously administered bDMARDs (P < 0.001).

### Drug retention

Overall, 455 treatment courses (43.9%) were stopped by 36 months. A total of 217 (20.9%) were stopped due to inefficacy, 113 (10.9%) due to non-toxic reasons [34 (3.3%) due to patient preference, 23 (2.2%) due to change in hospital, 56 (5.4%) due to other nontoxic reasons], 86 (8.3%) due to toxic reasons [34 (3.3%) due to infection, 28 (2.7%) due to other adverse events such as hematological, pulmonary, renal, or cardiovascular complications or malignancy, and 24 (2.3%) due to skin or systemic reaction], and 39 (3.8%) due to remission.

**Fig 1:**
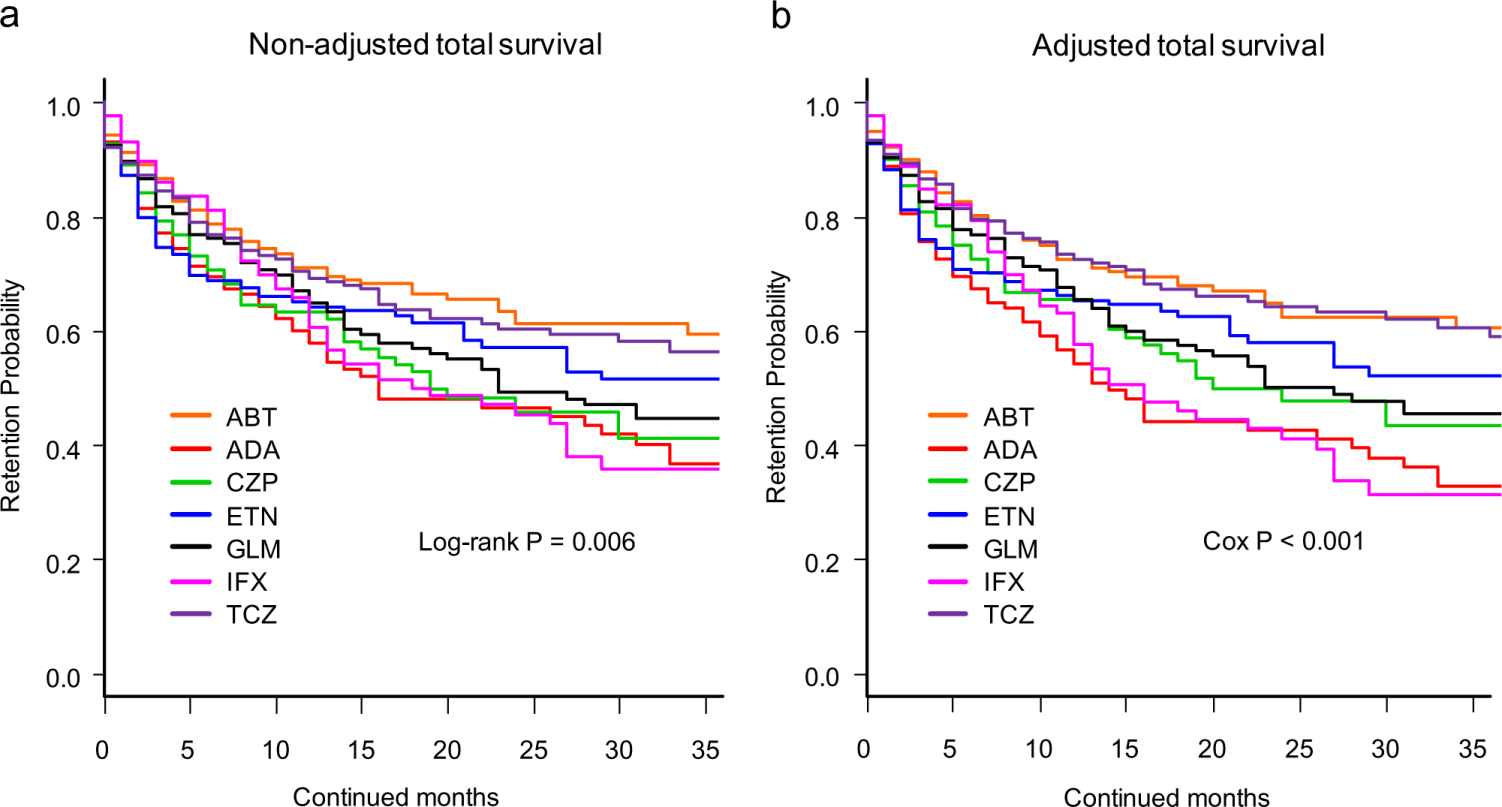
**Overall drug survival rates of (a) non-adjusted and (b) adjusted cases.** Adjusted confounder s were baseline sex, age, disease duration, DAS28-ESR, HAQ-DI, RF and ACPA positivity, concomitant MTX and PSL dose, presence of concomitant csDMARDs (BUC, IGU, SASP, and TAC), date of starting bDMARDs, and number of previously used bDMARDs.ABT = abatacept, ADA = adalimumab, CZP = certolizumab pegol, ETN = etanercept, GLM = golimumab, IFX = infliximab, TCZ = tocilizumab, DAS28-ESR = Disease Activity Score in 28 joints using erythrocyte sedimentation rate, HAQ-DI = Health Assessment Questionnaire disability index, RF = rheumatoid factor, ACPA = anti- cyclic citrullinated peptide antibody, MTX = methotrexate, PSL = prednisolone, csDMARDs = conventional synthetic disease-modifying antirheumatic drugs, BUC = bucillamine, IGU = iguratimod, SASP = salazosulfapyridine, TAC = tacrolimus, bDMARDs = biological disease-modifying antirheumatic drugs.

Total drug retention rates were analyzed using Kaplan-Meier estimates in both the non-adjusted model (Fig 1A) and adjusted model for potent cofounders using Cox proportional hazards regression modeling (Fig 1B). At 36 months, drug retention rates were as follows: 1) non-adjusted model: ABT (59.4%), ADA (36.8%), CZP (41.2%), ETN (51.6%), GLM (44.7%), IFX (35.7%), and TCZ (54.7%) (log-rank P = 0.006), and 2) adjusted model: ABT (60.7%), ADA (32.7%), CZP (43.3%), ETN (51.9%), GLM (45.4%), IFX (31.1%), and TCZ (59.2%) (Cox P < 0.001). Of note, treatment with ABT (Cox P = 0.0002) and TCZ (Cox P = 0.0009) showed significantly higher persistency compared to IFX in the adjusted model. Concerning other confounders, combined MTX dose (hazard ratio (HR) = 0.96, 95% confidence interval (CI) = 0.94–0.98, P = 0.0002) and TAC (HR = 0.63, 95%CI = 0.41–0.97, P = 0.036) at baseline showed positive effects, while combined PSL dose (HR = 1.02, 95%CI = 1.01–1.04, P = 0.0038), female sex (HR = 1.33, 95%CI = 1.06–1.69, P = 0.016), and number of previously administered bDMARDs (HR = 1.13, 95%CI = 1.04–1.22, P = 0.0031) at baseline showed negative effects on total drug retention.

### Causes of discontinuation

**Fig 2:**
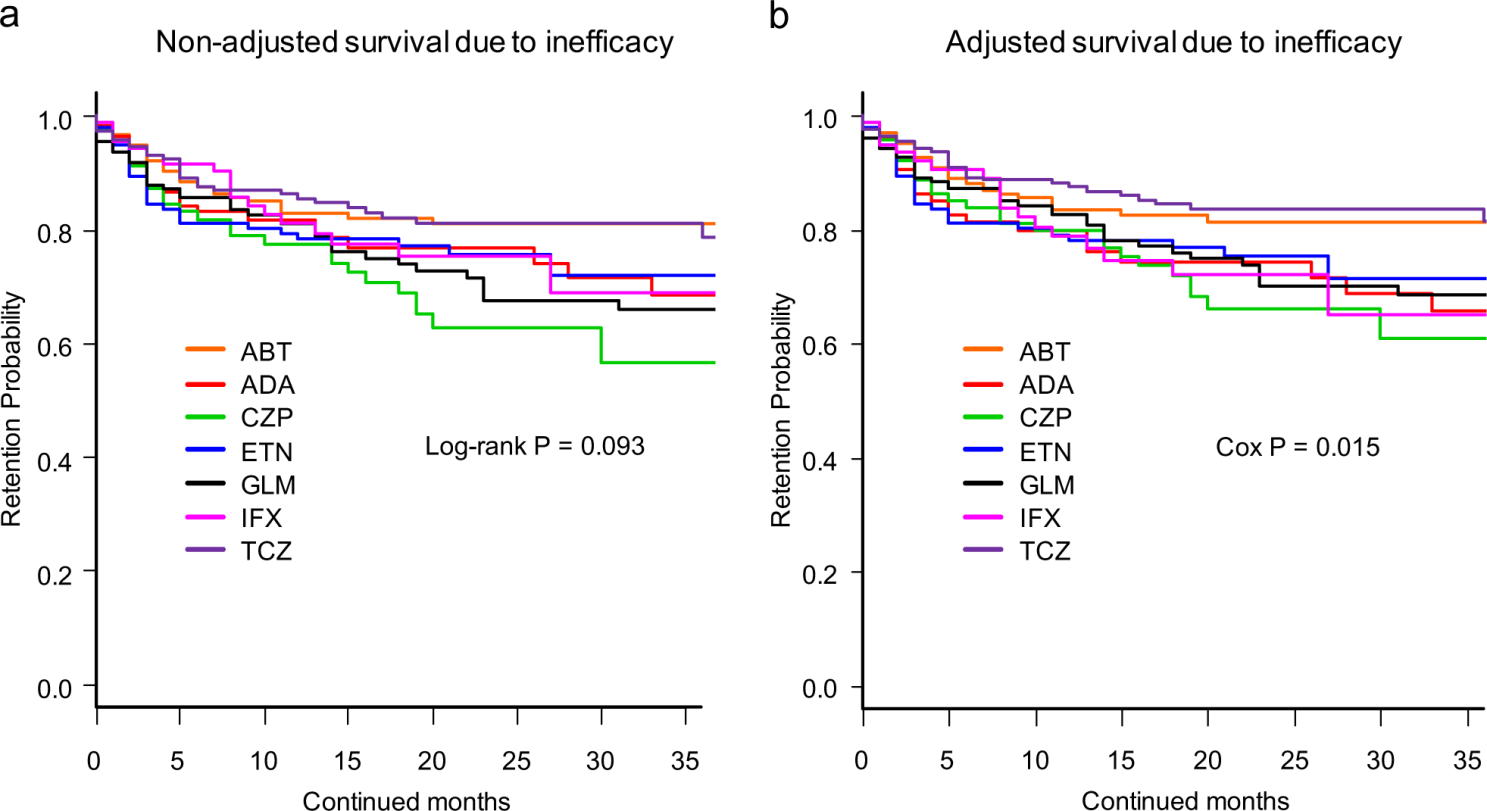
**Drug survival rates due to inefficacy of (a) non-adjusted and (b) adjusted cases.** Adjusted confounder s were baseline sex, age, disease duration, DAS28-ESR, HAQ-DI, RF and ACPA positivity, concomitant MTX and PSL dose, presence of concomitant csDMARDs (BUC, IGU, SASP, and TAC), date of starting bDMARDs, and number of previously used bDMARDs. ABT = abatacept, ADA = adalimumab, CZP = certolizumab pegol, ETN = etanercept, GLM = golimumab, IFX = infliximab, TCZ = tocilizumab, DAS28-ESR = Disease Activity Score in 28 joints using erythrocyte sedimentation rate, HAQ-DI = Health Assessment Questionnaire disability index, RF = rheumatoid factor, ACPA = anti- cyclic citrullinated peptide antibody, MTX = methotrexate, PSL = prednisolone, csDMARDs = conventional synthetic disease-modifying antirheumatic drugs, BUC = bucillamine, IGU = iguratimod, SASP = salazosulfapyridine, TAC = tacrolimus, bDMARDs = biological disease-modifying antirheumatic drugs.

**Fig 3:**
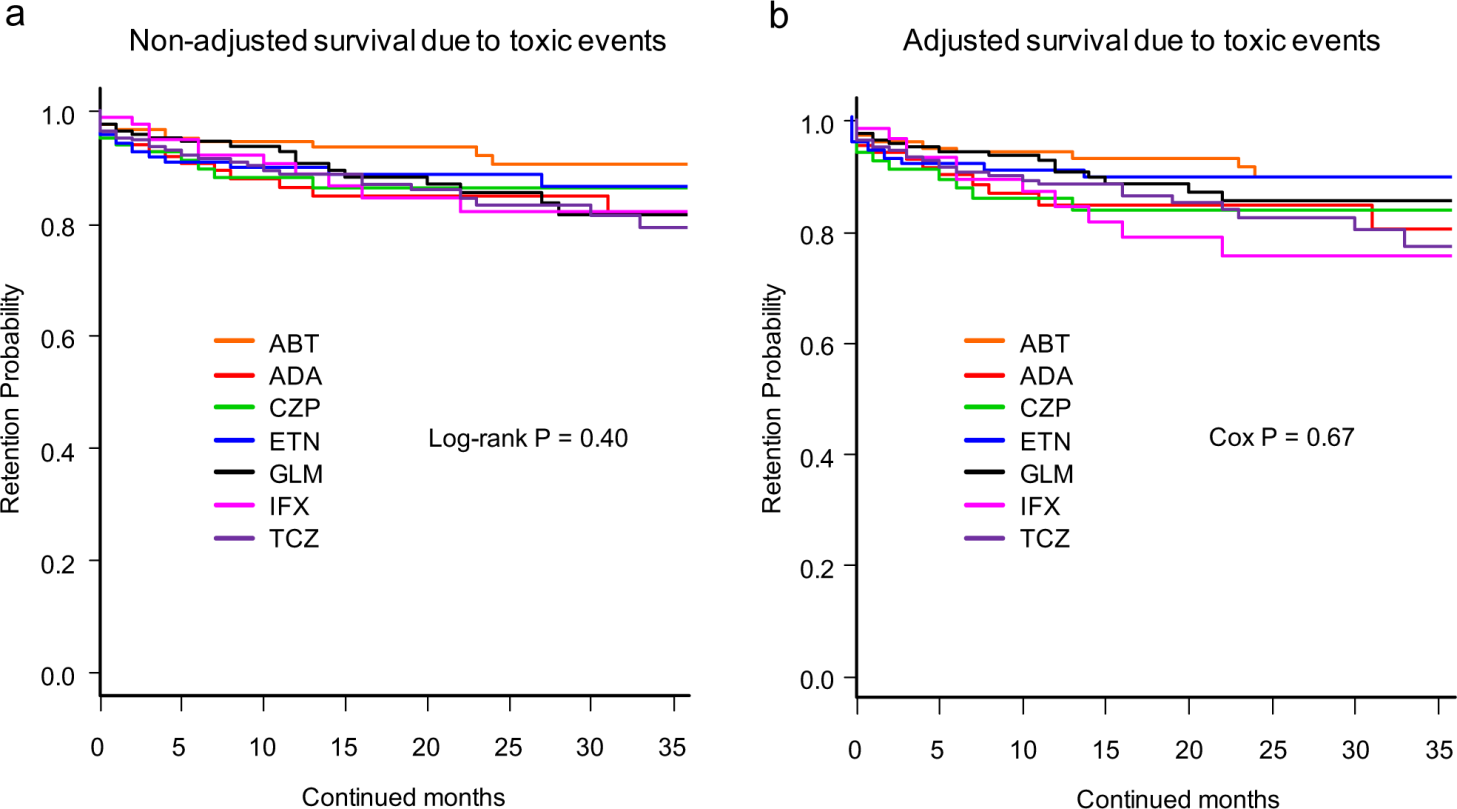
**Drug survival rates due to toxic adverse events of (a) non-adjusted and (b) adjusted cases.** Adjusted confounder s were baseline sex, age, disease duration, DAS28-ESR, HAQ-DI, RF and ACPA positivity, concomitant MTX and PSL dose, presence of concomitant csDMARDs (BUC, IGU, SASP, and TAC), date of starting bDMARDs, and number of previously used bDMARDs. ABT = abatacept, ADA = adalimumab, CZP = certolizumab pegol, ETN = etanercept, GLM = golimumab, IFX = infliximab, TCZ = tocilizumab, DAS28-ESR = Disease Activity Score in 28 joints using erythrocyte sedimentation rate, HAQ-DI = Health Assessment Questionnaire disability index, RF = rheumatoid factor, ACPA = anti- cyclic citrullinated peptide antibody, MTX = methotrexate, PSL = prednisolone, csDMARDs = conventional synthetic disease-modifying antirheumatic drugs, BUC = bucillamine, IGU = iguratimod, SASP = salazosulfapyridine, TAC = tacrolimus, bDMARDs = biological disease-modifying antirheumatic drugs.

**Fig 4:**
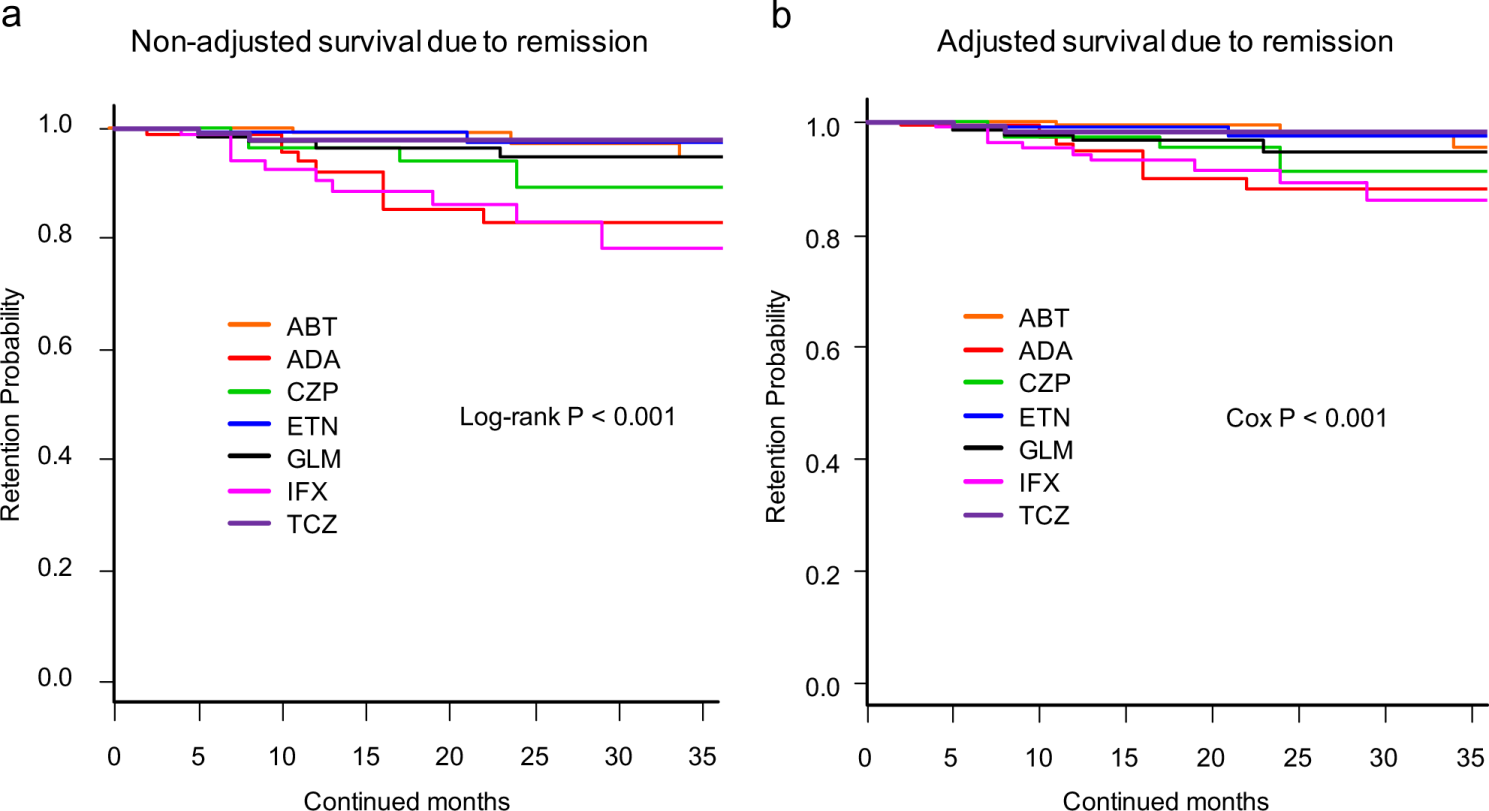
**Drug survival rates due to remission of (a) non-adjusted and (b) adjusted cases.** Adjusted confounder s were baseline sex, age, disease duration, DAS28-ESR, HAQ-DI, RF and ACPA positivity, concomitant MTX and PSL dose, presence of concomitant csDMARDs (BUC, IGU, SASP, and TAC), date of starting bDMARDs, and number of previously used bDMARDs. ABT = abatacept, ADA = adalimumab, CZP = certolizumab pegol, ETN = etanercept, GLM = golimumab, IFX = infliximab, TCZ = tocilizumab, DAS28-ESR = Disease Activity Score in 28 joints using erythrocyte sedimentation rate, HAQ-DI = Health Assessment Questionnaire disability index, RF = rheumatoid factor, ACPA = anti- cyclic citrullinated peptide antibody, MTX = methotrexate, PSL = prednisolone, csDMARDs = conventional synthetic disease-modifying antirheumatic drugs, BUC = bucillamine, IGU = iguratimod, SASP = salazosulfapyridine, TAC = tacrolimus, bDMARDs = biological disease-modifying antirheumatic drugs.

Cause-specific cumulative discontinuation rates were assessed using Kaplan-Meier estimates in both non-adjusted and adjusted models for potent cofounders using Cox proportional hazards regression modeling (Figs 2–4). At 36 months, drug retention rates due to inefficacy (Fig 2) were as follows: 1) non-adjusted model; ABT (81.0%), ADA (68.6%), CZP (56.5%), ETN (72.0%), GLM (65.9%), IFX (68.8%), and TCZ (78.6%) (log-rank P = 0.093) (Fig 2A); and 2) adjusted model; ABT (81.4%), ADA (65.7%), CZP (60.7%), ETN (71.3%), GLM (68.5%), IFX (65.0%), and TCZ (81.4%) (Cox P = 0.015) (Fig 2B).

Drug persistency rates due to all toxic adverse events (Fig 3) were as follows: 1) non-adjusted model; ABT (90.5%), ADA (81.3%), CZP (86.3%), ETN (86.6%), GLM (81.5%), IFX (81.9%), and TCZ (79.3%) (log-rank P = 0.40) (Fig 3A); and 2) adjusted model; ABT (89.8%), ADA (80.5%), CZP (83.9%), ETN (89.2%), GLM (85.5%), IFX (75.6%), and TCZ (77.2%) (Cox P = 0.67) (Fig 3B).

Drug persistency rates due to remission (Fig 4) were as follows: 1) non-adjusted model; ABT (94.7%), ADA (82.9%), CZP (89.5%), ETN (97.2%), GLM (94.7%), IFX (78.0%), and TCZ (98.0%) (log-rank P < 0.001) (Fig 4A); and 2) adjusted model; ABT (95.5%), ADA (88.1%), CZP (91.1%), ETN (97.5%), GLM (94.7%), IFX (86.4%), and TCZ (98.4%) (Cox P < 0.001) (Fig 4B). The number at risk of each bDMARD is shown in S1 Table.

Hazard ratios (HRs) of discontinuation due to each specific cause were calculated using multivariate Cox proportional hazards regression modeling (Table 2). HRs for discontinuation due to overall causes were significantly lower in ABT [HR = 0.50, 95%CI = 0.34–0.73, P<0.001] and TCZ (HR = 0.54, 95%CI = 0.37–0.79, P = 0.0014) compared to IFX, and significant differences were seen between the seven bDMARDs (P<0.001). In terms of HRs for discontinuation due to inefficacy, TCZ showed a significantly lower rate compared to IFX (HR = 0.56, 95%CI = 0.31–0.98, P = 0.043), and the difference was significant between the seven bDMARDs (P = 0.015). No significant difference was observed in HRs for discontinuation due to all toxic adverse events, including infection and systemic or skin reaction. However, ABT showed significantly lower HRs for other toxic events such as hematological, pulmonary, renal, cardiovascular complications and malignancy (HR = 0.24, 95%CI = 0.06–0.92, P = 0.037) compared to IFX, and the difference was significant between the seven bDMARDs (P = 0.0089). On the other hand, IFX showed higher HRs for remission compared to ABT (HR = 0.12, 95%CI = 0.03–0.45, P = 0.0015), ETN (HR = 0.14, 95%CI = 0.03–0.62, P = 0.0098), GLM (HR = 0.33, 95%CI = 0.11–0.98, P = 0.046), and TCZ (HR = 0.13, 95%CI = 0.03–0.46, P = 0.0017), and the difference was significant between the seven bDMARDs (P<0.001).

**Table 2:** Causes of treatment discontinuation at 36 months (Cox proportional hazards model, adjusted analysis).

	Reference	HR (95%CI)						
Variable	IFX	ABT	ADA	CZP	ETN	GLM	TCZ	P-value
	(n = 88)	(n = 221)	(n = 115)	(n = 82)	(n = 141)	(n = 175)	(n = 215)	
Total discontinuation events	1	0.50 (0.34–0.73)***	1.18 (0.81–1.73)	0.67 (0.44–1.02)	0.71 (0.48–1.05)	0.86 (0.59–1.24)	0.54 (0.37–0.79)**	<0.001
Inefficacy	1	0.69 (0.38–1.24)	1.05 (0.55–2.00)	1.02 (0.55–1.89)	0.99 (0.55–1.80)	1.21 (0.70–2.11)	0.56 (0.31–0.98)*	0.015
All toxic adverse events	1	0.53 (0.24–1.19)	1.06 (0.46–2.40)	0.77 (0.32–1.84)	0.73 (0.33–1.64)	0.85 (0.39–1.83)	0.90 (0.44–1.84)	0.67
Infection	1	1.22 (0.33–4.51)	0.89 (0.18–4.41)	0.87 (0.18–4.34)	1.59 (0.41–6.17)	0.61 (0.12–3.06)	0.71 (0.17–2.98)	0.78
Systemic or skin reaction	1	0.00 (0.00-infinite)	1.21 (0.34–4.37)	0.79 (0.19–3.25)	0.64 (0.16–2.60)	0.32 (0.06–1.77)	0.42 (0.10–1.69)	0.6
Other toxic events	1	0.24 (0.06–0.92)*	1.32 (0.37–4.71)	0.42 (0.09–1.88)	0.34 (0.08–1.41)	1.29 (0.42–4.03)	1.02 (0.33–3.13)	0.0089
Non-toxic reasons	1	1.23 (0.49–3.07)	1.50 (0.56–4.05)	0.40 (0.10–1.56)	1.17 (0.45–3.05)	0.79 (0.28–2.19)	0.73 (0.27–1.91)	0.37
Remission	1	0.12 (0.03–0.45)**	0.98 (0.40–2.41)	0.33 (0.10–1.04)	0.14 (0.03–0.62)**	0.33 (0.11–0.98)*	0.13 (0.03–0.46) **	<0.001

HR = hazard ratio; 95%CI = 95% confidence interval, IFX = infliximab, ABT = abatacept, ADA = adalimumab, CZP = certolizumab pegol, ETN = etanercept, GLM = golimumab, TCZ = tocilizumab.

The significance of differences was assessed using the Kruskal-Wallis nonparametric test for continuous variables and Pearson’s chi-square test for categorical variables.

* P<0.05

**P<0.01

*** P<0.001.

## Discussion

In this study, ABT and TCZ showed higher overall retention rates, TCZ showed lower inefficacy rates, and ABT showed lower toxic events (excluding infection and systemic or skin reaction) rates compared to IFX, while IFX showed higher discontinuation rates due to remission compared to ABT, ETN, GLM, and TCZ, after adjusting for potential confounders.

Concerning TNFi, previous reports have demonstrated that the largest reason for discontinuation was inefficacy (55.8%) [1], and ETN showed a higher retention rate compared to ADA and IFX [1, 3, 5], which correspond to our results.

With respect to biologics of non-TNFi, we have previously reported that TCZ and ETN showed higher retention, and TCZ showed lower inefficacy compared to ADA and IFX [23]. Kubo et al. showed that ABT and TCZ showed similar retention (ABT 72%, TCZ 69%) and remission rate (ABT 18%, TCZ 20%) after adjustment by propensity score matching at 52 weeks [24]. In addition, in TNFi failure patients, ABT and TCZ showed similar retention (ABT 54%, TCZ 64%) and a good-or-moderate EULAR response (ABT 77%, TCZ 84%) at 48 weeks [25]. Another report also showed that in patients with first TNFi failure, switching to non-TNFi-bDMARDs showed higher retention rate compared to switching to second-TNFi after adjustment for propensity scores [8]. Collectively, TCZ and ABT may exhibit higher retention rates compared to other TNFi in both bio-naïve and bio-switched patients in routine care.

In reference to treatment holiday due to remission of bDMARDs, previous reports have demonstrated that IFX and ADA seem to have better potential for discontinuation compared to CZP or ETN, as shown in the BeSt, HIT HARD, and OPTIMA studies in early RA, and in the RRR and HONOR studies in established RA [26–33], which agree with our result. However, these previous reports may have influenced the decisions regarding discontinuation by each physician, and further study is required to compare the maintenance of bDMARD-free remission between these agents.

Factors affecting bDMARDs retention and response have been reported. Female sex [5], concomitant PSL [3], high DAS28 or HAQ [3, 9, 34], absence or low dose of combined MTX [3, 9], and number of previous bDMARDs [9] were negative predictors, while concomitant use of csDMARDs besides MTX was a positive predictor of retention [5], which correspond with our results.

In this study, baseline DAS28-ESR and HAQ-DI did not show significant influences on total drug retention, maybe due to uniformity of these parameters between agents. On the other hand, combined dose of MTX and presence of TAC showed positive effects, while combined PSL dose showed negative effects on total drug retention in this study, suggesting the impact of these factors in both TNFi and non-TNFi retention.

Regarding to the efficacy of low-dose MTX in Japanese compared to Western populations, intraerythrocyte MTX-polyglutamate (MTX-PG) concentrations, which have been suggested as a useful biomarker of efficacy, reached 94 nmol/L at 10.3 mg/week of MTX in Japanese, compared to 65 nmol/L at 13.4 mg/week of MTX in the United States [35]. As a result, a relatively low dose of MTX may exhibit positive effects on bDMARDs retention in Japanese compared to Western populations. Previous studies have demonstrated that the efficacy of bDMARDs is enhanced by combination with csDMARDs such as BUC [15, 16], IGU [17], SASP [16, 18], and TAC [19, 20]. However, only TAC showed significant effects, and the effects of other csDMARDs were relatively marginal when adjusted by other confounders. Finally, bDMARDs retention in both non-adjusted and adjusted models by these possible confounders were evaluated. The tendencies were similar in both models in general, suggesting the predominance of the difference of bDMARDs in drug retention.

Some limitations to this study need to be considered. The number of patients was relatively small, as we only recruited patients who fulfilled the clinical backgrounds data, which may affect bDMARDs retention. However, this may also be a strength of this study. Second, the judgment and reasons for discontinuation depended on the decisions of each physician, without standardized criteria. Third, this was a retrospective study and the backgrounds of patients differed between the agents. Fourth, the minor dose changes of csDMARDs and PSL during the treatment period could not be monitored. However, the strength of this study was that treatment choice and discontinuation judgments were based on a real-world setting, and also the novelty of a trial to evaluate retention rates and discontinuation reasons for these seven bDMARDs.

## Conclusions

ABT and TCZ showed higher overall retention, TCZ showed lower inefficacy compared to IFX, while IFX showed higher discontinuation due to remission compared to ABT, ETN, GLM, and TCZ at 36 months when adjusted by potent confounders.

## Supporting information

S1 Table (DOCX)
