# Supplementary material for: Drug retention and discontinuation reasons between seven biologics in patients with rheumatoid arthritis -The ANSWER cohort study-
Source: PLoS One. 2018 Mar 15;13(3):e0194130. doi: 10.1371/journal.pone.0194130 (PMC5854351; doi:10.1371/journal.pone.0194130)
Supplement: S1 Table — (DOCX) [file pone.0194130.s001.docx]

**Number at risk of each biologic agent**

ABT = abatacept, ADA = adalimumab, CZP = certolizumab pegol, ETN = etanercept, GLM = golimumab, IFX = infliximab, TCZ = tocilizumab.
